# Interventions for preventing diarrhea-associated hemolytic uremic syndrome: systematic review

**DOI:** 10.1186/1471-2458-13-799

**Published:** 2013-09-03

**Authors:** Diana E Thomas, Elizabeth J Elliott

**Affiliations:** 1 Discipline of Paediatrics and Child Health, Sydney Medical School, University of Sydney, Sydney, Australia; 2 Centre for Evidence Based Paediatrics Gastroenterology and Nutrition (CEBPGAN), The Sydney Children's Hospitals Network (Westmead), Westmead NSW 2145, Australia; 3 Kids Research Institute, The Sydney Children's Hospitals Network (Westmead), Westmead NSW 2145, Australia

**Keywords:** Hemolytic uremic syndrome, HUS, Prevention, *E. coli* O157: H7, Public health

## Abstract

**Background:**

Hemolytic Uremic Syndrome (HUS) may follow infection with Shiga-toxin-producing organisms, principally *E. coli* O157: H7 (STEC), causing high morbidity and mortality. Our aim was to identify interventions to prevent diarrhea-associated HUS.

**Methods:**

Systematic search of the literature for relevant systematic reviews (SRs), randomised controlled trials (RCTs) and public health guidelines.

**Results:**

Of 1097 animal and 762 human studies, 18 animal studies (2 SRs, 2 reviews, plus 14 RCTs) and 6 human studies (3 SRs, plus 3 RCTs) met inclusion criteria. *E. coli* O157: H7 Type III secreted protein vaccination decreased fecal *E. coli* O157 shedding in cattle (P = 0.002). *E. coli* O157: H7 siderophore receptor and porin proteins (SRP) vaccines reduced fecal shedding in cows (OR 0.42 (95% CI 0.25 to 0.73) and increased anti-*E. coli* 0157: H7 SRP antibodies in their calves (P < 0.001). Bacterin vaccines had no effect. Probiotic or sodium chlorate additives in feeds reduced fecal *E. coli* O157 load as did improved farm hygiene (P < 0.05). Solarization of soil reduced *E. coli* O157: H7 contamination in the soil (P < 0.05). In an RCT examining the role of antibiotic treatment of *E. coli* O157: H7 diarrhea, HUS rates were similar in children treated with Trimethoprim-sulfamethoxazole and controls (RR 0.57; 95% CI 0.11 to 2.81). In another RCT, HUS rates were similar in children receiving Synsorb-Pk and placebo (RR 0.93; 95% CI 0.39 to 2.22). In one SR, hand washing reduced diarrhea by 39% in institutions (IRR 0.61; 95% CI 0.40 to 0.92) and 32% in community settings (IRR 0.68; 95% CI 0.52 to 0.90) compared to controls. Guidelines contained recommendations to prevent STEC transmission from animals and environments to humans, including appropriate food preparation, personal hygiene, community education, and control of environmental contamination, food and water quality.

**Conclusions:**

Animal carriage of STEC is decreased by vaccination and improved farm practices. Treatment of STEC diarrhea with antibiotics and toxin-binders did not prevent HUS. Public health interventions are the key to preventing STEC-associated diarrhea and HUS.

## Background

Diarrhea-associated Hemolytic Uremic Syndrome (HUS) usually affects young children and occurs sporadically or in outbreaks, as in Germany in 2011 [1]. HUS may complicate diarrhea due to Shiga-toxin-producing organisms including *Shigella dysenteriae* and Shiga-toxin-producing *E. coli* (STEC). Worldwide, STEC O157: H7 is the most common cause of HUS [1], although many serotypes have been implicated. In adults, STEC infections occasionally cause HUS, but more commonly cause thrombotic thrombocytopenic purpura (TTP) [2]. In HUS, renal thrombotic microangiopathy results in clinical presentation with acute renal impairment, thrombocytopenia and microangiopathic hemolytic anemia.

Although most patients with diarrhea-associated HUS recover from the acute episode, there is potential for long-term renal impairment and extra-renal complications including seizures, diabetes, severe colitis and hypertension, are common. In one study, 39% of participants with HUS had one or more abnormality at 10-year follow-up, including proteinuria, low creatinine clearance or hypertension [3]. In another study, 63% of children recovered fully while others had proteinuria, reduced creatinine clearance and/or hypertension and 3.4% developed end-stage renal failure. [4].

Outbreaks of STEC diarrhea are often traced to animals, particularly cattle. Approximately 30% of feedlot cattle shed *E. coli* O157: H7 [5]. Other animals [6,7]; contaminated water, both for drinking [8] and in swimming pools [9] and lakes [10]; food such as meats [11], mettwurst [12], salad sprouts [13] and lettuce [14]; drinks including unpasteurized apple juice [15] and milk; and direct contact with animals in petting farms [16] may also be sources of STEC.

In Australia and the USA the annual incidence of diarrhea-associated HUS in children under 5 years is ~1 per 100 000, with 3%-6% mortality [17,18]. HUS in the elderly causes death in up to 90% [19,20]. STEC 0157 infections cost the USA over U$400 million annually [21]. Approximately 8% of STEC infections progress to HUS [18]. Hence, prevention of HUS would significantly impact health outcomes and health expenditure.

Our aim was to systematically search and review the literature for SRs and RCTs of interventions to prevent diarrhea-associated HUS and to identify relevant evidence-based guidelines and public health policies.

## Methods

We performed electronic searches of CENTRAL (Issue 3, March 2012), Medline (1946 to March week 1, 2012) and EMBASE (1988 to 2012, week 11). For animal studies we searched Medline (1990-week 3, 2012). We used a search strategy, with no restriction on language, to identify relevant trials and systematic reviews (See Search Strategy, Additional file 1). We also reviewed reference lists of papers identified in the search. Electronic searches of the internet and medical literature were performed for evidence-based guidelines and public health policies addressing prevention or treatment of STEC infections to prevent HUS.

**Figure 1:**
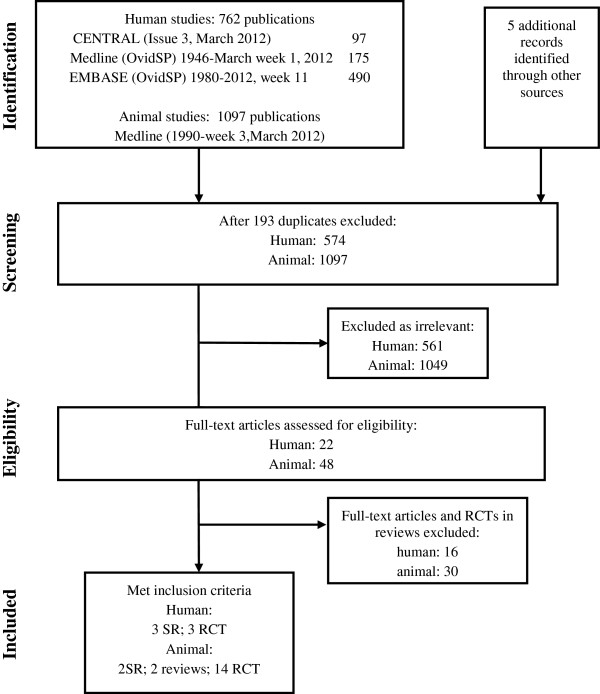
**PRISMA diagram**.

Eligible studies included RCTs for the prevention of STEC infections or diarrhea-associated HUS; SRs; evidence-based guidelines; and public health policies or recommendations on prevention of STEC infection and/or HUS. We included any intervention for preventing *E. coli* infection and/or HUS. Two reviewers independently reviewed abstracts obtained from the literature search to identify relevant publications (Figure 1) [22]. The quality of RCTs was assessed by two reviewers, based on specific criteria for minimizing bias, including sequence generation, allocation concealment, blinding, complete outcome data and selective outcome reporting [23,24].

## Results

We identified 1097 animal studies in the literature search, of which 18 (2 SRs [25,26], two reviews [5,27] and 14 additional RCTs [28-42]) met our inclusion criteria. One SR evaluated animal vaccination [30], another SR examined the effect of a wide range of farming practices on fecal *E. coli* O157 load [26] and two reviews evaluated dietary manipulation [5,27]. Of 762 human studies, six (3 SRs [43-45] and 3 additional RCTs [46-48]) met inclusion criteria. One SR investigated hand washing [45] and two SRs investigated specific treatments (antibiotics for STEC infection [43,44].

### Prevention of animal carriage

Two SR [25,26], 2 reviews [5,27] and 14 additional RCT [28-42] on prevention of animal carriage of *E. coli* O157 infections were included in the review. Potential ways to minimize animal carriage of STEC included: animal vaccination, additives and manipulation of animal feeds, and farm practices (Table 1).

**Table 1:** **Prevention of animal carriage of *E. coli *0157: H7**

**Study type**	**Study**	**Population**	**Intervention/Comparison**	**Results**
**Animal vaccination**				
SR	Pre-harvest interventions for *E. coli* 0157: H7 [26]	Domestic ruminants	Vaccines, probiotics, antimicrobials, chlorate treatment	*L. acidophilus NP51* and *P. freudenreichii* were the only interventions significantly proven in field trials to increase animal resistance to colonization with *E. coli* O157.
SR	Vaccinations to reduce faecal shedding of *E. coli* 0157: H7 [25]	Domestic ruminants	Type III secreted proteins of *E. coli* O157: H7 vaccination	Meta-analysis of eight comparisons showed a significant reduction in *E. coli* O157 faecal prevalence (OR = 0.38, 95% CI = 0.20, 0.51) [25] .
	Siderophore receptor and porin protein (SRP) vaccines [25]	Cattle	SRP vaccination	Meta-analysis of three trials showed significantly reduced *E. coli* O157 faecal shedding in cattle (OR = 0.42, 95% CI = 0.20,0.61) [25]. Although there was heterogeneity in the meta-analyses, these results indicate that type III and SRP protein vaccines decrease the faecal shedding of *E. coli* O157 in cattle [25] .
	Bacterin vaccines [25]	Cattle	Bacterin vaccination	No significant effect on *E. coli* O157 faecal shedding [25] .
RCT	Vaccination of calves [28]	60 calves	Vaccination with commercially produced type III secreted proteins (TTSP)/placebo	During peak shedding (days 3–6):
				Vaccinated animals showed mean log reduction of 1.4 (P = 0.002)
				Number of animals shedding significantly lower among the vaccinates (P ≤ 0.05)
RCT	Vaccination with *E. coli* 0157: H7 SRP bacterial extract [29]	437 cows from 2 commercial cow-calf herds	Vaccinated calves born to cows vaccinated pre-partum with *E. coli* 0157: H7 SRP vaccine/Placebo treatment	Calves born to vaccinated cows had significantly higher titres of anti *E. coli* 0157: H7 SRP antibodies in circulation at branding time (P < 0.001)
**Diet**				
SR	Pre-harvest interventions [26]	Ruminants	Food additives e.g. probiotics, sodium chlorate, antimicrobials, bacteriophages	Probiotics and sodium chlorate were effective in reducing *E. coli* O157 load. Antimicrobials neither decreased nor increased load. There was insufficient evidence to support the effectiveness of bacteriophages and other food additives. The review was limited by lack of high quality studies, inconsistent results and conduct of studies in an artificial environment.
R	Diet, *E. coli* 0157: H7 and cattle [5]	Cattle	Alterations in diet and dietary supplements, forage, grains, hay	Grains can increase fecal *E. coli* 0157: H7 shedding. *E. coli* 0157: H7 concentrations in cattle can be affected by the composition of their diet.
R	Forage feeding interventions to reduce preharvest *E. coli*[49]	Cattle	Forage feeding compared to grain-based feeds	Change in diet from grain (corn) to forage (hay) decreased faecal *E. coli* O157: H7 in cattle by up to 1000-fold within 5 days
RCT	Dried distiller’s grains (DG) [30]	28 pens of feedlot cattle	Dried DG or dry rolled corn/2 × 2 factorial design	No significant effect of DG or dry rolled corn on *E. coli* 0157: H7 prevalence
RCT	Wet DG with solubles (WDGS) [31]	603 feedlot steers	40% WDGS/No WDGS	Higher levels of faecal *E. coli* 0157: H7 in WDGS-fed animals compared to controls (P < 0.001), indicating WDGS feeding may increase *E. coli* 0157: H7 levels
RCT	Wet DG [32]	272 feedlot crossbred beef heifers	Addition of Wet DG to feed/no WDG	No significant effect of feeding wet DG on faecal *E. coli* 0157: H7
RCT	WDGS [34]	608 heifers	Animals fed 40 or 70% WDGS, then switched to 0 or 15% for 56 days/no WDGS	Significantly lower *E. coli* 0157:H7 in faeces and on hides after lowering levels of dietary WDGS (≤ 15% for last 56 days prior to harvest (P < 0.05)
**Probiotics**				
SR	**Probiotics** (6 RCTs) [26]	Ruminants	Probiotics/No treatment or control	Evidence of efficacy for probiotic combination *L. acidophilus* NP51 (NPC 747) and *P. freudenreichii*
RCT	Microbial feedings [35]	Cattle	Microbial feedings	Decreased *E. coli* O157 on cattle hides (74% less likely, P < 0.05) and 69% less likely in faeces (P < 0.01)
RCT	Probiotics [36].	448 steers	*L. acidophilus* NP51/control	Steers fed *L. acidophilus* NP51 were 35% less likely to shed *E. coli* 0157: H7 than steers in untreated pens (OR = 0.58, P = 0.008)
RCT	Probiotics [37]	Cattle	*L. acidophilus* NP51	Cattle on *L. acidophilus* NP51 excreted less *E. coli* 0157 in the feeding period compared with the controls (P < 0.01), Dose response was a linear decrease in excretion with increasing dose (P < 0.01)
**Bacteriophage**				
RCT	Bacteriophage [38]	Cattle	Poly-encapsulated phages/control	Poly-encapsulated phages did not reduce duration of shedding (P < 0.1)
**Vitamin D**				
RCT	Vitamin D [39]	Two groups of Cattle (beef and dairy)	Received .5 × 10 (6) IU vitamin D per day for 10 days/Control treatment	No significant effect on faecal shedding of *E. coli* 0157: H7
**Other purpose supplements**				
RCT	Feed supplements of Monensin, Ractopamine [40]	720 crossbred beef steers	Monensin (33 or 44 mg/kg of DM)	Significantly less faecal *E. coli* 0157: H7 with monensin at 44 mg/kg of feed, than at 33 mg/kg (4.3 vs 6.8%, P = 0.05). Urea or ractopamine had no effect (P = 0.9)
			Urea (0, 0.35, 0.70% of DM) or ractopamine (0,200 mg/steer)/2 × 3 ×2 factorial block design	
**Farm practices**				
RCT	Young cattle – farm practices [41]	57 farms	Multiple interventions were applied to 3 groups of farms of young animals. Group A: No new animals, no contact with other cattle and no shared water sources; bedding and animals kept dry; animals kept clean; animals kept as a closed group; boot-dip and overcoat used. Group B: No new animals, no contact with other cattle and no shared water sources; water troughs emptied and cleaned weekly. Group C received all the interventions of A and B.	The effect of each intervention was analysed by univariable comparative analysis. Dry bedding and retaining animals in original groups were the most important measures of the intervention s (P < 0.05)
			Control farms had no alteration in practices	
RCT	Solarization of soil in feedlot pen to reduce *E. coli* 0157: H7 [42]	Feedlot pen divided into 40 plots	Soil solarization in feedlot pen surface material/No solarization	>3.0-log reduction of *E. coli* 0157: H7 by week 6 of solarization (P < 0.05) in the treated pens compared to control

RCT randomised controlled trial; SR systematic review; R non-systematic review.

#### Vaccination of animals

Two SR [25,26] and two additional relevant RCT [28,29], not included in the SR, investigated animal vaccination. One RCT investigated *E. coli* O157 Type III secreted protein (TTSP) vaccines [28] and the second RCT investigated *E. coli* O157: H7 siderophore receptor and porin protein (SRP) vaccines [29]. A systematic review on the role of vaccines in reducing fecal shedding of *E. coli* O157 in weaned domestic ruminants was inconclusive. Two studies reported a significant reduction of *E. coli* O157 shedding after *E. coli* O157 TTSP vaccination (P < 0.05) [26] but study protocols were heterogeneous. In a more recent systematic review with meta-analysis, *E. coli* O157: H7 TTSP vaccines significantly reduced fecal load of *E. coli* O157 in cattle (OR 0.38 95% CI 0.29 to 0.51) [25]. *E. coli* O157: H7 SRP vaccines reduced fecal load (OR 0.42 (95% CI 0.20 to 0.61), but bacterin vaccines were ineffective [25]. Thus, RCTs in domestic ruminants (cattle and pigs) showed that some vaccines increase protective antibody levels and decrease colonization, carriage, duration of fecal shedding and transmission of STEC. In a recent RCT, calves vaccinated with an *E. coli* O157 TTSP vaccine shed significantly less *E. coli* O157 than the control group on days 3–10 after vaccination (P = 0.002). Also, the number of calves shedding *E. coli* O157 during days 3–6 was significantly lower among the vaccinated, compared to control group (P ≤ 0.05) [28]. There was a low risk of bias in this study: interventions were randomly assigned and there was allocation concealment and blinding of staff to treatment. Another study reported that calves born to cows vaccinated with *E. coli* O157: H7 SRP vaccine had higher titers of anti- *E. coli* O157: H7 SRP antibodies at branding time (P < 0.001) [29]. However *E. coli* O157: H7 SRP vaccination had effect on fecal *E. coli* O157 shedding (P > 0.05), indicating that timing of vaccination may be relevant [29]. Treatments were randomly assigned, there was allocation concealment, and both study and laboratory personnel were blinded, indicating low risk of bias in this study.

#### Animal feeds and supplements

We identified one SR [26], two reviews [5,49] and nine RCT [30-32,35-37,40,50,51] not included in the reviews evaluating manipulation of animal diets. Interventions included addition of probiotics (SR [26], plus 3 additional RCT [35,37,50]), grain versus forage feeds (2 reviews [5,26], plus 4 RCT [30-32,51]), and feed supplementation with vitamin D (1 RCT [36]), chlorate (SR) [27] and other additives (1 RCT [40]). The SR examined the effect of a wide range of farming practices on fecal *E. coli* O157 load [26]. Addition of probiotics and sodium chlorate to feed or water was effective in reducing fecal *E. coli* O157 load, but antimicrobials neither decreased nor increased the load [26]. There was insufficient evidence on the effectiveness of bacteriophages and other feed additives such as oral polyclonal anti-*E. coli* O157: H7 antibodies to support their use. The review was limited by lack of high quality studies, inconsistent results, and conduct of studies in an artificial environment. One RCT not included in the SR showed improved hygiene (dry bedding and cohorting of animals by herd) decreased fecal shedding of *E. coli* O157 (P < 0.05) [41]. A recent RCT concluded that soil solarization in feeding pens significantly decreased *E. coli* O157: H7 contamination of soil (P < 0.05) [42].

#### Probiotics

One SR of preharvest strategies [26], and 3 additional RCTs [35-37] reported on probiotics. The SR included 6 RCTs, in which controls received no treatment or placebo [26] and concluded that there was evidence of efficacy for the probiotic combination *L. acidophilus* NP51 (NPC 747) and *P. freudenreichii*[26] in reducing fecal STEC. A 10-year review reported that STEC could be significantly reduced by probiotics [5].

After microbial feeds, *E. coli* O157 was 74% less likely to be isolated from hides of cattle (P < 0.05) and 69% less likely to be found in faeces (P < 0.01) [35]. Steers fed *L. acidophilus* NP51 were 35% less likely to shed *E. coli* 0157: H7 than controls (OR 0.58, P = 0.008), confirming results from earlier trials [36]. Another RCT showed that cattle given *L. acidophilus* NP51 shed fewer *E. coli* 0157 in the feeding period compared with controls (P < 0.01), there being a dose response with a linear decrease in *E. coli* O157 shedding with increasing probiotic dose (P < 0.01) [37]. One SR (with 1 RCT and 3 challenge trials) concluded that addition of sodium chlorate to feed significantly decreased fecal shedding of *E. coli* O157: H7 [26].

#### Distillers’ grains

Two reviews [5,49] and four additional RCTs [30-32,34] reported on the effect of dietary distillers’ grains (DG) on fecal shedding. Up to 30% of cattle, particularly feedlot cattle, shed *E. coli* O157: H7 [5]. To increase feed efficiency, cattle are fed high grain rations [49], such as distillers’ grains (DG), which consist mainly of bran, protein and germ [52]. Grain-fed cattle have increased fecal shedding of *E. coli* O157: H7 because of alterations in gut fermentation, suggesting that dietary manipulations and feed supplements may affect ruminal or hindgut fermentation of DG and alter fecal shedding of *E. coli* O157: H7 [5,33,40]. An abrupt change in diet from grain (corn) to (forage) hay decreased fecal *E. coli* O157: H7 load in cattle by up to 1000-fold within 5 days, suggesting this intervention could be used pre-slaughter to reduce *E. coli* contamination [49]. In a large scale RCT however, no association was found between *E. coli* 0157: H7 load and feeding dried distiller’s grains, in contrast to previous trials [30]. A RCT in feedlot cattle confirmed that feeding wet DG had no effect on fecal *E. coli* O157: H7 load [32]. In another RCT wet DG with solubles (WDGS) increased fecal *E. coli* O157: H7 shedding in cattle (P < 0.001) [31] and lowering the dietary WDGS concentration for 56 days prior to slaughter reduced shedding (P < 0.05) [34].

#### Vitamin D

Vitamin D supplementation in cattle naturally infected with *E. coli* 0157: H7 does not affect shedding of *E. coli* 0157: H7 [39].

#### Bacteriophages

A SR concluded there was insufficient evidence of effectiveness of bacteriophages on fecal shedding [26]. A recent RCT investigated bacteriophages adapted to *E. coli* O157: H7 and encapsulated to prevent inactivation by gastric acid. These bacteriophages did not reduce shedding of *E. coli* O157: H7 in feedlot cattle [38], although they reduced duration of shedding by 14 days compared to controls (P < 0.1) [38].

#### Antibiotics, antimicrobials and growth promoters

One SR [26] and 2 RCTs [40,53] investigated these food additives. No study included in the SR found a significant association between ionophore use and fecal shedding of *E. coli* O157 [26]. One RCT investigated the effect of two commercial feed additives, monensin (an ionophore against gram-positive bacteria) and ractopamine (a growth promoter) on fecal *E. coli* O157: H7 [40]. Cattle fed monensin at 44 mg/kg of feed had less fecal *E. coli* O157: H7 than cattle fed 33 mg/kg (4.3 vs. 6.8%, P = 0.05) [40]. Ractopamine had no effect on fecal shedding of *E. coli* O157: H7 (4.4 vs. 4.0%, P = 0.89) [40]. In one RCT *B. subtilis* was ineffective in reducing *E. coli* O157: H7 in feed lot cattle [53].

#### Farm practices

Two relevant RCTs evaluating farm practices were identified [41,42]. One investigated whether improved hygiene and farm practices prevented carriage of *E. coli* O157: H7 [41]. Thirty farms (all having young cattle), were divided into four groups. Interventions were introduced to change farm practices in three groups of farms, which were compared with the fourth (control) group. Interventions included providing a clean environment and/or closed groups of young stock, improving water and feed hygiene, or no change in practice (controls). Providing dry bedding and keeping animals in the same herd groupings significantly decreased *E. coli* O157: H7 levels during the 4.5 month trial (P < 0.05) [41]. Treatments were randomly assigned with the allocation concealed; however blinding was not possible due to the differences in the treatments, so risk of bias is unclear. In a recent RCT, solarization of soil in feedlot pens was investigated, because soil is a potential point at which to reduce *E. coli* O157: H7 contamination [42]. After 1 week of solarization, there was a 2.0-log decrease in *E. coli* in soil and by week 6 of solarization, there was a >3.0-log reduction (P = 0.05) compared to controls [42].

### Treatment of human STEC infections

#### Minimization of person to person contact and isolation of infected persons

During STEC outbreaks in the community or hospitals, people infected should be isolated to contain the outbreak [54]. Infections that may be complicated by HUS may be spread through person-to-person contact [55] in institutions (including psychiatric hospitals [56], nurseries [57], and child care centers [58]) and from mother to baby [59]. Guidelines to minimize person-to-person transmission have been issued by the Health Protection Agency (HPA) in the UK for public health physicians and environmental health officers. They recommend microbiological screening of close contacts of infected persons, provision of adequate hygiene and toilet facilities and supervised hand washing, particularly with children [60,61]. UK guidelines advise that the best way for patients or their carers to avoid STEC infection is to adhere to essential hygiene, including frequent hand washing with soap and water, touching the face only with clean hands and keeping hard surfaces clean and disinfected [62].

An appropriate public health response to the management of acute bloody diarrhea in children is an important first step in preventing HUS. Children with proven STEC infection should not return to school, nursery or childcare until 48 hr after diarrhea ceases. Longer exclusion periods may be necessary for children aged less than five or with difficulty following hygienic practices.

During an outbreak of STEC diarrhea, UK guidelines recommend [63]:

•Immediate risk assessment of the school’s eating/food preparation areas and toilets

•Exclusion of high risk children until microbiological clearance

•Exclusion of symptomatic children and staff until microbiological clearance

•Consideration of microbiological screening and exclusion of asymptomatic contacts when there is a risk of ongoing environmental or person-to-person transmission

•Improvement in hand hygiene within the institution e.g. increasing supervision of hand washing by children, particularly those under 5 years

•Provision of information to parents on measures to control spread, including improved hygiene at home

•Implementing environmental controls such as increased cleaning and decontamination of high risk surfaces, toys, toilets and eating facilities

•Improving food preparation procedures where necessary

•Consideration of temporary school closure if there is a risk of ongoing transmission from the environmental or person-to-person

•Searching for additional cases in the community and consider collecting and analyzing epidemiological data and/or environmental samples from the school

•Ensuring appropriate levels of hygiene are maintained according to guidelines and that there are effective procedures to remedy non-compliance

•Considering measures for long term infection control, such as altering the layout of wash basins and toilets, or providing more such facilities in prominent positions [63]

#### Inpatient management

Inpatients with STEC infections should be isolated to prevent spread of infection to other patients, staff and visitors. Fluids should be monitored and the patient observed for complications, including signs of HUS [1]. Anti-diarrheal and anti-motility agents should be avoided [1,64]. STEC infection should be notified to the relevant public health authority and patient contacts assessed.

#### Antibiotics

The risk of developing HUS after antibiotic treatment for *E. coli* O157: H7 diarrhea in children was addressed in two SRs [43,44]. Both were inconclusive: antibiotics neither prevented nor increased the risk of HUS. Panos reviewed 19 studies with no meta-analysis, concluding that more RCTs were required to determine the effect of antibiotics on the duration and severity of enteritis and the potential for developing HUS [44]. Safdar included nine heterogeneous studies (only one RCT) [43]. Six of these studies showed no increased risk of HUS associated with antibiotic use in STEC infection; one showed a protective effect and two showed an increased risk. However, meta-analysis showed neither protection nor increased risk of HUS with antibiotic use (pooled odds ratio 1.15 (95% CI 0.79-1.68). We identified two completed RCTs, which examine the role of specific treatments for STEC in preventing HUS [46,47] (Table 2).

**Table 2:** Interventions for the prevention of HUS in human STEC infection

**SR/RCT**	**Population**	**Intervention**	**Outcome**	**Results**		
**Systematic reviews**						
Safdar 2002 [43] 9 studies	Patients with *E. coli* 0157: H7 enteritis, some developing HUS	Antibiotics	Development of HUS	Meta-analysis showed neither protection nor increased risk of HUS with antibiotics. Pooled odds ratio 1.15 (95% CI 0.79-1.68)		
Panos 2006 [44] 19 studies with no meta-analysis	Patients with *E. coli* 0157: H7 enteritis	Antibiotics	Development of HUS and duration/severity of enteritis	Inconclusive: concluded that more studies were required to determine effect of antibiotics on duration and severity of enteritis, and development of HUS		
**Randomised controlled trials**				**AR of HUS**	**AR reduction (NNT to prevent HUS)**	**Relative risk**
				**Treatment vs control**		**(95% CI)**
Proulx 1992 RCT [46]	Children with diarrhoea and *E. coli* 0157: H7 isolated in stool. Mean age 64 months (range 3–213 months). N = 47 (Intervention group n = 22; no treatment n = 25)	Trimethoprim-sulfamethoxazole (4/20 mg/kg/dose) twice daily for 5 days versus no antibiotics	Development of HUS	9% vs 16%	7%; (14)	0.57
						(0.09-3.46)
						P = 0.67
Rowe 1997 [47]	Children with documented *E coli* 0157 (or other STEC) infection, close contact with HUS or STEC infection; or symptoms of STEC infection N = 353	Synsorb-Pk versus placebo	Development of HUS:	5% vs 5.6%	0.6% (167)	0.93
			1. All included patients (n = 353)			(0.39-2.22)
			2. Patients with proven STEC infection (n = 119)	12% vs 15%	3% (33)	0.76
						(0.30-1.92)
			3. Patients treated <4 days from diarrhoea onset (n = 73)	11% vs 25%	14% (7)	0.46
						(0.15-1.35)
Taylor 2011[48]	Children 6 months to 18 years presenting with bloody diarrhea at 13 clinical centres in South America (still enrolling)	Infusion of Shigamabs, monoclonal antibodies against Shiga-toxin 1 and Shiga-toxin 2, versus placebo	Safety, tolerability, efficacy, pharmacokinetics	Trial in progress	Taylor 2011 [48]	Children 6 months to 18 years presenting with bloody diarrhea at 13 clinical centres in South America (still enrolling)

RCT randomised controlled trial; SR systematic review; AR absolute risk; NNT number needed to treat; CI confidence interval.

#### TM (trimethoprim-sulfamethoxazole, TMP-SMX) antibiotic

In the RCT [46] included in Safdar’s SR [43], TMP-SMX for 5 days was compared with no antibiotic in children with diarrhea in who tested positive for *E. coli* O157: H7 (N = 47). There is a potential risk of bias in this trial: although it was randomized using computer generation, allocation concealment was not specified, neither participants nor investigators were blinded and use of intention-to-treat analysis was not stated. There was 100% follow-up and no adverse effects were reported. The HUS rate in children treated with antibiotics did not differ statistically from controls (RR 0.57; 95% CI 0.09 to 3.46, P = 0.67). Treatment commenced only after *E. coli* O157: H7 was isolated from stools, so there was potentially a delay between diarrhea onset and antibiotic treatment. At randomization only 20% of participants were excreting *E. coli* O157: H7 in the stool, hence if the infection was already established, the pathogenic mechanisms leading to HUS may already have been triggered prior to antibiotic treatment.

#### Synsorb-Pk

In the second completed RCT, Synsorb-Pk (SP) treatment for 7 days was compared with corn meal (control) in children with *E. coli* O157: H7 infection, symptoms consistent with STEC infection, or a close contact with HUS or STEC infection [47]. Synsorb is an agent that binds to ST to prevent its absorption from the gut. There is a risk of bias in this RCT because the methods of randomization and allocation concealment were not specified and intention-to-treat analysis was not stated. Both participants and investigators were blinded. No adverse effects were reported. The risk of developing HUS in the SP and control groups was similar RR 0.93 (95% CI 0.39 to 2.22). Thus, there is no evidence that Synsorb-Pk is effective in preventing HUS secondary to STEC infection. In this trial only 20% of participants were enrolled within 4 days of onset of diarrhea. Thus, Shiga toxin may already have been absorbed from the gut in the majority, rendering ineffective a treatment designed to bind and prevents absorption of toxin.

#### Monoclonal antibodies

An RCT currently in progress is assessing the role of monoclonal antibodies against Shiga-toxins 1 and 2 in preventing HUS in children with STEC infection [48].

### Environmental contamination

#### Open farms, petting zoos and farms

To minimize environmental contamination in wildlife open or pet farms, provision of adequate hand washing facilities and eating areas that are separate from the animals is essential [45,63]. Following outbreaks of *E. coli* infection involving children who visited petting farms, the Centers for Disease Control (CDC) and Prevention in the USA published guidelines that included recommendations to prevent veterinarians, farm visitors, animal keepers and visitors from acquiring infection in petting zoos and other public farms [66]. The most effective intervention is hand washing [63,66]. Animals should be kept healthy, visitors should be informed about measures to prevent catching diseases from animals, food should be prohibited in areas where animals are kept, and areas for food consumption should be separated from animals by a transition area [66]. The Health Protection Agency (HPA) UK specifically designed guidelines for the general public when visiting farms, following an outbreak of *E. coli* O157 infection [67]. They also advise that the risk of acquiring serious *E. coli* O157 infection from animals and their surroundings will be greatly reduced if people maintain basic hygiene and warn that pregnant women should be particularly careful. The main recommendations are that people should avoid touching their face or put fingers in mouths after patting animals or visiting farms. Children in particular should not put their faces close to, or kiss, animals. There should be no eating or drinking while walking round the farm or touching animals. No food that drops on the ground should be eaten. Soap and water should be used to clean hands because this is more effective at removing *E. coli* O157 in dirt than gels or wipes. Children should be well-supervised to ensure they wash their hands thoroughly. Hands should be washed after touching animals or surfaces such as fences and before eating or drinking. Eating should only occur in separately located, designated areas such as restaurants or picnic areas. After contact with animals, boots, shoes, clothes and prams should be carefully cleaned, with thorough hand washing to follow [67].

#### Illustrative case 1. Petting zoo

***Outbreak:****159 cases of E. coli O157: H7 infection in children who attended a fair in Canada in 1999*[68]*.*

***Detection:****After interviewing people testing positive for E. coli 0157: H7 or with diarrhea, authorities identified animals from the travelling petting zoo as a potential source of infection, since goats and sheep from this zoo, but no local cows, tested positive for E. coli 0157: H7. The same rare phage type was identified by subtyping in both human and animal samples.*

***Public health response:****Guidelines developed as a result of this outbreak recommended separation of animals and food outlets and better facilities for hand washing after contact with animals*[63]*, consistent with other studies*[45]*.*

#### Swimming water

HUS has been linked to contamination of private [69] and public [70] paddling pools with *E. coli* O157 and may result from failure to drain pools after use or inadequate chlorination [70]. WHO guidelines for safe swimming pools and other recreational waters *‘Water sanitation and health’* provide recommendations to ensure water hygiene for public swimming pools and lakes, including monitoring and surveillance of water quality, cleanliness of the facility, and measures to reduce infection risk, including education, design and construction of facilities and high quality maintenance and operation [71]. The guidelines target national and local authorities, owners, operators and designers, public health professionals, researchers and the general public [71]. Other guidelines regarding water quality of pools and lakes include recommendations for disinfecting pools [72], managing health and safety [73] and operating facilities [74]. Wildlife such as ducks are a potential source of *E. coli* contamination in lakes [75]. Guidelines such as those from the National Health and Medical Research Council of Australian include procedures to help prevent contamination of swimming water [76]. Beach water and sand are potential sources of *E. coli* infection [77,78]. Health risk from sand is due mainly to contaminated sand on the hands being transferred to the mouth, so thorough hand washing may prevent infection [45].

#### Drinking water

Globally, one of the most critical determinants of human health is drinking water quality [71]. Diarrhea is the most common waterborne disease and over 4 billion diarrhea cases and over 2 million deaths occur globally each year [71]. Water may be contaminated in catchment areas by human or animal feces or within the supply system through unsanitary equipment or bad hygiene. Contaminated well water has been implicated in outbreaks of *E. coli* O157: H7 infection. In 1999, nearly 1000 people were infected and at least two died after consuming water at a county fair in New York State. The ground water well at the fair had been contaminated by manure run-off from a cattle exhibit barn after a heavy rainfall and the unchlorinated water was used by vendors for drinks and ice [8].

#### Illustrative case 2. Contaminated drinking water

***Outbreak:****In 2000 in Walkerton Ontario, Canada, 2,300 people became infected with E. coli O157: H7 in contaminated drinking water, of whom 65 were hospitalized and seven died*[79]*.*

***Detection:****A judicial provincial government inquiry found numerous contributing failures, including assumptions that bore water was safe, insufficient surveillance of bore catchment areas, inadequate chlorination of water and inadequately trained operating staff in the treatment plant*[79]*.*

***Public health response:****Following the outbreak, new Drinking Water Regulations were introduced for providers, including stringent guidelines for testing for bacterial contamination*[79]*. Canadian guidelines now recommend regular testing of all drinking water systems for E. coli. The number, frequency, and location of samples depends on the type and size of the system and local authority requirements*[80]*. The maximum acceptable concentration of E. coli in public, semi-public, and private drinking water systems is set at non-detectable (0 mg/L)*[80]*.*

Guidelines for improving water quality were amongst the earliest produced by the World Health Organization (WHO) and are regularly revised [71]. Many countries including Australia produce their own guidelines for monitoring drinking water quality [81]. Many guidelines originated in response to outbreaks of *E. coli* infection.

### Domestic food preparation: prevention of contamination

Public education regarding safe preparation of domestic food is important. The WHO produces guidelines on preparing food for the general public [65,82].

The five keys to safer food are:

1. Keep clean (hands, food preparation equipment and kitchen areas);

2. Separate raw and cooked food (e.g. keep raw meat from salad foods; use separate equipment such as knives and chopping boards for raw food; store raw food in separate containers to avoid cross contamination);

3. Cook and reheat food thoroughly, especially meat, poultry, eggs and seafood, to ensure food reaches boiling temperature ( ≥70°C) to kill *E. coli.*;

4. Store food at safe temperatures (refrigerate promptly, don’t leave cooked food at room temperature for > 2 hours, don’t store food for too long, don’t thaw frozen food at room temperature, and keep cooked food hot at > 60°C) and;

5. Use safe water and raw materials (fresh food, washed fruit and vegetables especially if eaten raw, in-date food, food processed for safety e.g. pasteurized milk [65].

### Public health prevention measures

Over 700,000 guidelines were identified in the internet search using text words ‘guidelines’ , ‘*E. coli* O157′ and ‘prevention’. We selected representative, evidence-based guidelines, such as those from the Centers for Disease Control, USA which summarize strategies for preventing infection with *E. coli* O157: H7 and other STEC. Prevention measures include appropriate food preparation and storage, promotion of personal hygiene, public health education campaigns, legislation, programs to prevent environmental contamination by STEC, and testing food and water quality.

#### Hand washing

There is strong evidence that hand washing is effective in preventing diarrhea, hence indirectly reducing the risk of HUS infection. A Cochrane review included 14 RCTs [45]: eight institution-based trials in high income countries, five community-based trials in lower income countries, and one in high risk (AIDS) patients. Meta-analysis showed hand washing lowered diarrhea rates by approximately one third: in institutions (2 RCTS, 39% reduction, IRR 0.61 (95% CI 0.40 - 0.92); and in community settings (4 RCTS, 32% reduction, IRR 0.68 (95% CI 0.52 - 0.90) [45]. Hand washing guidelines are available from WHO [82] and elsewhere [83-85]. The WHO recommends frequent, thorough washing with soap and water, lathering for at least 20 seconds [82]. Hand sanitizers should be used where soap and water are unavailable [82]. The Mayo Clinic notes that antibacterial soap is no more effective for killing pathogens than ordinary soap and may lead to bacterial resistance [83]. Hands should be washed before preparing food, eating, attending to the sick or injured; and after preparing raw meat and poultry, using the toilet, changing babies, touching animals or their accoutrements, blowing noses, treating wounds or the sick, and handling contaminated waste [83-85]. Children should be supervised when hand washing [83].

### Commercial food production: control to minimize *E. coli* contamination

The Food and Agricultural Organization (FAO) has produced guidelines to assist countries strengthen national systems to ensure food safety and quality [86]. These guidelines also ensure that exported/imported food comply with the international regulations relevant in a globalized food industry. As consumers take more interest in the provenance of their food, nations develop guidelines and regulations to minimize contamination during production. The FAO guidelines provide recommendations to protect public health and prevent food adulteration through improvement of food control systems, including legislation and infrastructure [86]. These can be adapted by individual countries to their own circumstances. The European Community (EC) established the European Food Safety Authority (ESFA) to protect food safety by providing general requirements for laws on food. Since food is freely transported within the EC, it was essential to ensure that legislation was consistent throughout the EC [87].

Potential contamination of food by STEC may occur at any stage in the production chain – including contamination of seeds (a particular problem with salad sprouts) or of foodstuffs during packing, transport and distribution [88]. Hence, microbiological testing of food for *E. coli* is recommended at all relevant stages of production. Because there have been more than ten serious outbreaks of food poisoning connected with salad sprouts in the USA, the US Food and Drug Administration classifies alfalfa sprouts as a high-risk food. The definitive way to remove EHEC during food production is by bactericidal treatment including cooking, pasteurization or irradiation [89] (Table 3).

**Table 3:** **Source of infection and strains of *E coli *identified in EHEC outbreaks**

**Source of infection**	**EHEC**	**Year**	**Location**
	**Strain**		
**Contaminated foods**			
*Meat:*			
Hamburger mince	O157: H7	1994	USA [11]
Hamburger mince	O157: H7	2009	USA [90]
Hamburger mince	O157: H7	2008	USA [91]
Hamburgers	O157: H7	2007	USA [92]
Beef	O157: H7	2009	USA [93]
Beef	O157: H7	2009	USA [94]
Mettwurst	O111: H_	1995	Australia [12,59]
Unknown	O111	1994	Italy [95]
Deer jerky	O157: H7	1997	USA [6]
Pepperoni (on Pizza)	O157: H7	2007	USA [96]
*Salad:*			
Sprouts	O104: H4	2011	Germany, France [97]
Radish sprouts in school lunches	O157	1996	Japan [13]
lettuce	O157: H7	1998	USA [14]
Lettuce (shredded)	O145	2010	USA [98]
Spinach	O157: H7	2006	USA [99]
*Dairy foods:*			
Unpasteurized cheese (made from goat and cow milks)	verotoxin VT2 gene	1992	France [100]
Cheese curds	O157: H7	2000	USA [101]
Cheese	O157: H7	2010	USA [51]
Raw Biscuit Dough	O157: H7	2009	USA [102]
**Contaminated drinks**			
Water	O157: H7	2000	Canada [79]
Water (from well)	O157: H7	1999	USA [8]
Unpasteurized apple juice (commercial)	O157: H7	1996	Canada; USA [103]
Unpasteurized milk	O157: H7	1997	USA [104]
Unpasteurized milk	O157	1999	UK [105]
Unpasteurized goat’s milk	O157: H7	1997	Bohemia [29,106]
Raw milk	O157	2008	USA [107]
**Contaminated environment**			
Petting zoo	O157	2002	Canada [68]
Petting zoo	O157	2000	The Netherlands [16]
Farm open to public	O157	1997	UK [108]
Farm	O157	2001	Scotland [109]
Farm: Dairy and petting farm	O157: H7	2003	USA [110]
Swimming (pool)	O157: H7	1991	USA [9]
Swimming (lake)	O157: H7	1999	USA [111]
Swimming (lake)	O121: H19	1999	USA [10]
**Person to person**			
Children via paddling pool	O157: 49	1992	Scotland [69]
Children via paddling pool	O157: 2	1993	UK [70]
Child care center	O157	1999	UK [58]
Day care nursery	O157	1995	UK [57]
Mother to baby	O111: H_	1998	Australia [59]
Institution	O157: H7	1990	USA [56]

#### Illustrative case 3. Contaminated Sprouts in Japan

***Outbreak***: *A large outbreak of EHEC O157 gastroenteritis occurred in Japanese school children, in 1996*[13]*. Over 12,000 children became ill and 121 developed HUS of whom three died.*

***Detection:****When epidemiological evidence implicated hydroponic radish sprouts in school lunches, laboratory analysis of sprout samples identified E. coli O157: H7 contamination*[13]*. Investigations could not confirm the source of contamination. After additional outbreaks in 1997 involving radish sprouts, it was found that the seeds were the source of contamination. On further investigation, sprouts grown from seeds or sprout roots experimentally contaminated with E. coli O157: H7, were found to be contaminated with E. coli O157: H7 both on outer surfaces and in inner tissues, due to rapid bacterial growth during germination.*

***Public health response:****To prevent future outbreaks, the Japanese Government developed new guidelines and regulations on growing, processing and shipping of radish sprouts. Their focus was on sterilization of both the water supply and the seeds. They also developed protocols for maintaining hygienic facilities and equipment and for examining radish sprouts for EHEC*[112]*. Guideline additions tightened regulations on production of hydroponically grown lettuce*[112].

#### Illustrative case 4. Contaminated Sprouts in Germany 2011

***Outbreak:****The 2011 outbreak in Germany of STEC infection resulted in over 4000 cases of severe gastroenteritis and over 850 cases of HUS, with 49 deaths.*

***Detection:****After investigating cases, salad greens were identified as the potential source of infection, cucumbers from Spain having been incorrectly implicated initially. Epidemiological investigation implicated a 15000 kg shipment of fenugreek seeds, imported from Egypt in 2009 for growing sprouts, as the probable source of the outbreak which affected mainly adults,*[97]*, even though the lethal STEC 0101:H4 involved was not detected on farms. The high proportion of cases progressing to HUS may reflect the STEC’s virulence characteristics*[113]*.*

***Public Health response:****The EC banned importation of fenugreek seeds from Egypt and the UKHPA updated guidelines on the management and treatment of acute bloody diarrhea in children, particularly in relation to STEC infection*[114]*.*

#### Meat and meat products

Many STEC outbreaks are related to meat products, particularly beef from feedlot cattle, so improving the safety of meat after slaughter by controlling *E. coli* O157: H7 contamination is an active areas of research [115].

#### Dry cured salami

Outbreaks of *E. coli* O157: H7 infection, occurring in Washington and California in1994, were traced back to commercially distributed dry cured salami. In response, the USA Centers for Disease Control and Prevention issued specific advice on preventing recurrence [116].

#### Illustrative case 5. Contaminated Mettwurst

***Outbreak:****In Australia in 1995, an outbreak of HUS caused by E. coli O111:H1 resulted in 21 HUS cases, with one death. Eighteen children required dialysis and complications included colonic necrosis, cerebral hemorrhage, convulsions and death. After one year, five children had renal function impairment*[59]*.*

***Detection:****Within two weeks the dietary habits of patients were investigated*[117]*. Patient samples and suspect foods were tested, using Shiga-like toxin gene assays. The infection source was rapidly identified as a locally produced dry-fermented sausage (mettwurst)*[12]*. Samples from 19 of the 21 HUS patients and 7 of 8 mettwurst samples collected from their homes tested positive for the organism.*

***Public health response****: The Australia New Zealand Food Authority updated legislation on the production of fermented, uncooked comminuted meat products, requiring that the products be cooked at a core temperature of 65°C for 10 minutes during production, so as not to rely on consumers cooking the products at home*[118]*.*

#### Hamburgers

American guidelines give specific advice on preparation of hamburgers, aimed at decreasing outbreaks of *E. coli* O157 infection from ingestion of contaminated mincemeat [11,91]. Mince or hamburgers should reach an internal temperature of 70°C to be safe to ingest, regardless of color. To ensure this temperature is reached, a food thermometer should be used, since color is not a sufficiently reliable indicator that harmful bacteria such *E. coli* O157: H7 have been killed. Eating undercooked hamburger mince poses a significant risk that can lead to serious illness or even death, particularly in the young, the aged, and people with immune deficiency. Under-cooked hamburgers should be sent back to the kitchen and served with a new bun on a clean plate. The 2007 CDC guidelines reinforce recommendation from the WHO and FAO.

#### Venison

Wildlife meat, such as deer, wild boar and hare, have been identified as a reservoir and potential source of pathogenic STEC infections [119]. Venison, which may be highly contaminated with fecal bacteria, is usually hung at ambient temperatures, allowing bacteria present to multiply [6]. In contrast, fresh beef is generally chilled rapidly. To protect public health, guidelines for handling game should similar to those for commercially slaughtered meat [120]. The US Department of Agriculture's Food Safety (USDA) and Inspection Service monitors commercial production of jerky, a dehydrated meat product [120]. US guidelines were also formulated for domestic production of jerky, following an outbreak of *E. coli* O157 infection in 11 people who ate homemade venison jerky [6]. The USDA recommends pre-cooking the meat to 70°C before dehydration to ensure decontamination.

#### Guidelines for the retail food industry

Despite guidelines, there continue to be outbreaks of STEC infection and HUT that can be traced back to contaminated food. Outbreaks sometimes result in development of new guidelines or revision of recommendations. Examples include revision of the food production code for mettwurst sausage following an HUS outbreak in Australia [12] and guidelines developed for businesses from Food Safety Australian [121] and others in the USA [122] and UK [123].

#### Illustrative case 6. Contaminated spinach in USA

***Outbreak:****In 2006, a large E. coli O157: H7 outbreak in the USA was associated with contaminated baby spinach. This caused 205 cases of diarrhea and three deaths: 51% of those infected were hospitalized and 16% developed HUS*[50]*.*

***Detection:****Government agencies determined bagged spinach to be the probable source of the outbreak. Contaminated spinach bags were collected from patients, and the packaging plant which processed the bags. Using bag product codes and DNA fingerprinting of bacteria from the bags, the outbreak strain was eventually matched to environmental E. coli O157: H7 samples from one agricultural field. Potential contamination sources of this field included wild pigs, irrigation wells and waterways exposed to cattle and wildlife feces. Due to the many potential sources of contamination, including animals, humans, and water, the way in which the E. coli O157: H7 contaminated the spinach was never elucidated.*

***Public health response:****New guidelines were developed to minimize microbial contamination during processing of fresh-cut produce*[124]*.*

Guidelines on preventing and controlling *E. coli* O157 infections are updated as new scientific evidence emerges. Recommendations include ways to prevent acquisition and spread of infection, especially within institutions where cross infection is more likely to occur and for people at increased risk of acquiring and transmitting infection, including food handlers and the young, elderly and infirm [125]. Preventative measures also include public health and education campaigns about food preparation and storage, commercially and at home; legislation applicable to food production; prevention of environmental contamination; and programs for monitoring food and water quality, including swimming facilities. Revisions are often instigated after investigations of HUS outbreaks.

The CDC’s advice can be summarized in five points. Wash hands thoroughly after: the bathroom, changing babies, contact with animals and their environment; and before preparing or eating food. Cook meat thoroughly to a minimum internal temperature of 70°C. Avoid unpasteurized milk, dairy products and juices such as fresh apple cider. Avoid swallowing water from swimming pools, paddling pools, lakes or rivers. Wash hands, surfaces, boards and utensils after preparing raw meat to prevent cross contamination [126].

## Discussion

Our aim was to review the medical literature and public health guidelines regarding prevention of diarrhea-associated HUS. We identified avenues for prevention of STEC infection and HUS, based on high quality evidence from SRs and RCTs. In animals, vaccination [28] and improved farming and feeding practices including dietary manipulation (e.g. probiotics and sodium chlorate feed additives [26]), cohorting of animals and dry bedding and soil solarization reduce STEC carriage and fecal shedding in animals, and hence the risk of STEC transmission to humans.

Appropriate treatment of STEC diarrhea in humans, including withdrawal from school and isolation within institutions will minimize spread of infections. There is strong evidence that personal hygiene, particularly hand washing, is crucial to prevent acquisition and spread of STEC infections in the community, hence decreasing the risk of HUS [45].

Public health guidelines and legislation to safeguard food and water against STEC contamination and ensure appropriate production, preparation and storage of food are also important, as is community education. Treatment of STEC diarrhea with antibiotics and Shiga-toxin binding agents does not influence risk of developing HUS, based on limited evidence from RCTs.

Despite evidence to support these prevention strategies and numerous evidence-based guidelines and policies, outbreaks of STEC diarrhea and HUS continue to occur. These are often traced to farms and particularly cattle, which are a major source of STEC infection, since ruminant animals act as a natural reservoir of *E. coli* 0157: H7 [27]. Thus strategies to reduce fecal *E. coli* O157: H7 shedding in cattle could reduce STEC infection in humans. *E. coli* 0157: H7 TTSP and SRP vaccines are effective in reducing fecal *E. coli* O157 shedding [28,127]. However, cattle infected with *E. coli* O157 are not ill, hence subsidies for farmers may be required to promote vaccination as a public health measure to decrease the risk of animal to human transmission. Vaccinating the majority of cattle in a feedlot pen also provides herd immunity [128]. If *E. coli* O157: H7 carriage and shedding can be reduced in cattle, environmental contamination will also be reduced, as will contamination of meat during processing [129].

Changes in gut fermentation may also affect fecal *E. coli* O157 shedding, since *E. coli* O157 multiplies rapidly in the gut [130]. Hence, altering intestinal conditions with different feeds has been investigated as a means of reducing fecal *E. coli* 0157: H7 shedding [131]. Some studies show that reducing WDGS feeds 56 days prior to harvest significantly reduces fecal shedding of *E. coli* 0157: H7 [34] and there is evidence that cattle fed hay rather than grain for a brief period before slaughter have significantly reduced fecal shedding of pathogenic *E. coli*[132]. However, other researchers have found no relation between *E. coli* 0157: H7 shedding and feed type [30].

Some *STEC* strains from cattle have developed antibiotic resistance and in one study resistance was found in 34% fecal samples from animals and also in isolates from hamburger mince [133]. Research is required to address antibiotic resistance.

Education about the risk factors for STEC infection, methods for rapidly identifying the infection source to prevent further spread, and isolation of symptomatic children and adults with STEC infection may help contain outbreaks and limit potential cases of HUS. In one RCT, treatment with a Shiga toxin binding agent was delayed until STEC was isolated in the stool, often days after onset of symptoms. Rapid, reliable diagnostic tests for STEC would enable early initiation of treatment and supportive care.

Appropriate management of STEC infections, once acquired, may also decrease the risk of developing HUS, including monitoring of fluid balance, early use of parenteral volume expansion to minimize renal damage [90], monitoring for complications and avoiding anti-diarrheal and anti-motility agents [1,64]. There is insufficient evidence to warrant use of antibiotics, dialysis or Shiga-toxin binders for early treatment of STEC infection, although these have been proposed as measures to prevent progression from STEC diarrhea to HUS. Indeed there is some concern about the potential harm associated with antibiotic treatment of STEC diarrhea. In a prospective cohort study, antibiotic treatment of STEC infections increased the risk of developing HUS and this has changed clinical practice for treatment of enteritis [134]. Participants in the study were children with confirmed stool *E. coli* O157: H7 and the RR for developing HUS in the antibiotic treated group compared to controls was 17.3 (95% CI 2.2-137, P = 0.007). In 1996 425 Japanese children hospitalized during a huge outbreak of STEC infection were treated with antibiotics. Although 12 children developed HUS, all recovered without significant complications [13]. Antibiotic treatment of *E. coli* infection [134] also introduces the potential for development of antibiotic resistance [133,135].

This is the first systematic review on this topic include both the human and animal literature and public health guidelines. It provides useful information for clinicians and public health professionals on the prevention of STEC infection and HUS. High quality evidence from RCTs and SRs is available to inform guidelines and medical and veterinarian practice. This value of the review is limited by the low number of relevant RCTs and their small sample size, which restricts our ability to draw meaningful conclusions.

Because HUS is a rare event, large multi-centered trials are required to provide sufficient power address some controversial questions, including the role of antibiotics for the treatment of STEC in the prevention of HUS. Outcome measures should including the frequency and severity of HUS and adverse effects of treatment and study power must be sufficient power to detect real benefits or harms. We eagerly await an ongoing RCT evaluating the role of monoclonal antibodies in inactivating Shiga-toxins 1 and 2 in children with STEC diarrhea [48]. Studies are also required to investigate the role of new antibiotics, new vaccines and new therapies for binding Shiga-toxin. The development of a human vaccine to prevent STEC infection may eventually provide herd immunity and protect against HUS and would be particularly valuable in low-income settings where bacterial gastroenteritis is common.

## Conclusions

Potential means of preventing HUS include minimizing fecal STEC shedding in animals and transmission of infection to humans. Public health measures to safeguard food and water from contamination are essential as is personal hygiene and care with food preparation and storage. This is the first comprehensive review of this topic and includes data from the medical and veterinarian literature and from public health guidelines. It provides useful information for clinicians and public health professionals on the prevention of HUS.

## Abbreviations

EHEC: Enterohemorrhagic *E. coli*; HUS: Hemolytic uremic syndrome; STEC: Shiga-toxigenic *E. coli*; CCTR: Cochrane Controlled Trials Register; TM: Trimethoprim-sulfamethoxazole; OR: Odds ratio; NNT: Number needed to treat; CI: Confidence intervals; SRP: Siderophore receptor and porin proteins; TTSP: Type III secreted protein; VTEC: Verotoxin-producing *E. coli*.

## Competing interests

The authors declare that they have no competing interests.

## Authors’ contributions

Both authors drafted and wrote the manuscript, revised it critically for intellectual content and read and approved the final manuscript. Both authors read and approved the final manuscript.

## Authors’ information

DET: Scientific Director, Centre for Evidence-Based Paediatrics Gastroenterology and Nutrition, Paediatrics and Child Health, Sydney Medical School,

The University of Sydney; KRI, The Children's Hospital at Westmead

EJE: Professor, Pediatrics and Child Health, Sydney Medical School,

The University of Sydney; Consultant Pediatrician, The Children's Hospital at Westmead; Director, Centre for Evidence-Based Paediatrics Gastroenterology and Nutrition

## Pre-publication history

The pre-publication history for this paper can be accessed here:

http://www.biomedcentral.com/1471-2458/13/799/prepub

## Supplementary Material

Additional file 1 Search strategy Click here for file
