# Supplementary material for: Interventions for preventing diarrhea-associated hemolytic uremic syndrome: systematic review
Source: BMC Public Health. 2013 Sep 3;13:799. doi: 10.1186/1471-2458-13-799 (PMC3844431; doi:10.1186/1471-2458-13-799)
Supplement: Additional file 1 — Search strategy [file 1471-2458-13-799-S1.docx]

### Additional file 1 – Search strategy

MEDLINE

HUS and Cochrane Precise RCT filter

1. Hemolytic-Uremic Syndrome/

2. hemolytic ur?emic syndrome.tw.

3. haemolytic ur?emic syndrome.tw.

4. Purpura, Thrombotic Thrombocytopenic/

5. thrombotic thrombocytop?enic purpura.tw.

6. or/1-5

7. randomized controlled trial.pt.

8. controlled clinical trial.pt.

9. randomized.ab.

10. placebo.ab.

11. clinical trials as topic.sh.

12. randomly.ab.

13. trial.ti.

14. or/7-13

15. exp animals/ not (humans/ and exp animals/)

16. 14 not 15

17. and/6,16

EMBASE

HUS and Cochrane EMBASE rct filter

1. Hemolytic uremic syndrome/

2. hemolytic ur?emic syndrome.tw.

3. haemolytic ur?emic syndrome.tw.

4. thrombotic thrombocytopenic purpura/

5. thrombotic thrombocytop?enic purpura.tw.

6. or/1-5

7. crossover-procedure/

8. double-blind procedure/

9. randomized controlled trial/

10. single-blind procedure/

11. random*.tw.

12. factorial*.tw.

13. (crossover* or cross-over*).tw.

14. placebo.tw.

15. ((doubl* adj blind*) or (singl* adj blind*)).tw.

16. assign*.tw.

17. allocat*.tw.

18. volunteer*.tw.

19. or/7-18

20. 6 and 19

CENTRAL search strategy

ID Search

#1 (h*emolytic next ur*emic syndrome):ti,ab,kw

#2 (thrombotic next thrombocytop*enic next purpura):ti,ab,kw

#3 (#1 OR #2)
